# Supplementary material for: Baseline Characterization of the Gut Microbiota of Field and Colony Populations of Phlebotomus tobbi and Preliminary Assessment of the Anti-Leishmanial Activity of Cultivable Bacteria
Source: Pathogens. 2026 Jun 23;15(7):658. doi: 10.3390/pathogens15070658 (PMC13416292; doi:10.3390/pathogens15070658)
Supplement: Supplementary file 1 [file pathogens-15-00658-s001.zip › pathogens-4357168-File S1/Taxa2ASV_Decomposer/Family/f__Burkholderiaceae/seqs.align.html]

```
seq6            AGTGGGGAATTTTGGACAATGGGcGaAAgCCTGATCCAGCAATGCCGCGTGTGTGAAGAA
seq7            AGTGGGGAATTTTGGACAATGGGgGcAAcCCTGATCCAGCAATGCCGCGTGTGTGAAGAA

seq6            GGCCTTCGGGTTGTAAAGCACTTTTGTCCGGAAAGAAAtgGCtcTGGTTAATACCtGgGG
seq7            GGCCTTCGGGTTGTAAAGCACTTTTGTCCGGAAAGAAAacGCcgTGGTTAATACCcGtGG

seq6            tcGATGACGGTACCGGAAGAATAAGgACCGGCTAACTACGTGCCAGCAGCCGCGGTAATA
seq7            cgGATGACGGTACCGGAAGAATAAGcACCGGCTAACTACGTGCCAGCAGCCGCGGTAATA

seq6            CGTAGGGTcCAAGCGTTAATCGGAATTACTGGGCGTAAAGCGTGCGCAGGCGGTtgtgcA
seq7            CGTAGGGTgCAAGCGTTAATCGGAATTACTGGGCGTAAAGCGTGCGCAGGCGGTccgctA

seq6            AGACcGATGTGAAATCCCCGaGCTTAACtTGGGAAtTGCATTgGTGACTGcacGGCTAGA
seq7            AGACaGATGTGAAATCCCCGgGCTTAACcTGGGAAcTGCATTtGTGACTGgcgGGCTAGA

seq6            GTgTGtCAGAGGGGGGTAGAATTCCACGTGTAGCAGTGAAATGCGTAGAGATGTGGAGGA
seq7            GTaTGgCAGAGGGGGGTAGAATTCCACGTGTAGCAGTGAAATGCGTAGAGATGTGGAGGA

seq6            ATACCGATGGCGAAGGCAGCCCCCTGGGatAAcACTGACGCTCATGCACGAAAGCGTGGG
seq7            ATACCGATGGCGAAGGCAGCCCCCTGGGccAAtACTGACGCTCATGCACGAAAGCGTGGG

seq6            GAGCAAA
seq7            GAGCAAA
```
